# Molecular Characterization of the Oncogene BTF3 and Its Targets in Colorectal Cancer

**DOI:** 10.3389/fcell.2020.601502

**Published:** 2021-02-11

**Authors:** Hantao Wang, Junjie Xing, Wei Wang, Guifen Lv, Haiyan He, Yeqing Lu, Mei Sun, Haiyan Chen, Xu Li

**Affiliations:** ^1^Department of Colorectal Surgery, Changhai Hospital, Shanghai, China; ^2^Department of Digestive Endoscopy, Changhai Hospital, Shanghai, China; ^3^Department of Anesthesiology, Changhai Hospital, Shanghai, China; ^4^Department of Endocrinology, Changzheng Hospital, Shanghai, China

**Keywords:** MiR-497, TP53, HERC2, basic transcription factor 3 (BTF3), colorectal cancer, CHD1L

## Abstract

Colorectal cancer (CRC) is one of the most commonly diagnosed and leading causes of cancer mortality worldwide, and the prognosis of patients with CRC remains unsatisfactory. Basic transcription factor 3 (BTF3) is an oncogene and hazardous prognosticator in CRC. Although two distinct functional mechanisms of BTF3 in different cancer types have been reported, its role in CRC is still unclear. In this study, we aimed to molecularly characterize the oncogene BTF3 and its targets in CRC. Here, we first identified the transcriptional targets of BTF3 by applying combined RNA-Seq and ChIP-Seq analysis, identifying CHD1L as a transcriptional target of BTF3. Thereafter, we conducted immunoprecipitation (IP)-MS and E3 ubiquitin ligase analysis to identify potential interacting targets of BTF3 as a subunit of the nascent-polypeptide-associated complex (NAC). The analysis revealed that BTF3 might also inhibit E3 ubiquitin ligase HERC2-mediated p53 degradation. Finally, miRNAs targeting BTF3 were predicted and validated. Decreased miR-497-5p expression is responsible for higher levels of BTF3 post-transcriptionally. Collectively, we concluded that BTF3 is an oncogene, and there may exist a transcription factor and NAC-related proteolysis mechanism in CRC. This study provides a comprehensive basis for understanding the oncogenic mechanisms of BTF3 in CRC.

## Introduction

Colorectal cancer (CRC) is one of the most commonly diagnosed and leading causes of cancer mortality worldwide (Bray et al., 2018). Although extensive progress has been made in chemotherapy, radiotherapy, immunotherapy, and surgery, the prognosis of patients with CRC is still unsatisfactory (Miller et al., 2016). Genetic and epigenetic molecular alterations at the transcriptional and post-transcriptional level are related to CRC occurrence, progression, treatment resistance, and patient outcomes (Keum and Giovannucci, 2019; Jung et al., 2020; Nguyen et al., 2020). A deeper understanding of the molecular mechanisms of CRC will provide novel insights into the pathogenesis of the disease and identify new treatment options.

Basic transcription factor 3 (BTF3), also known as nascent-polypeptide-associated complex (NAC) beta (NACB), was reported to serve as an oncogene and convey worse prognosis in gastric cancer (Liu et al., 2013; Zhang et al., 2017), pancreatic cancer (Kusumawidjaja et al., 2007), osteosarcoma (Liu et al., 2019a), cervical cancer (Wu et al., 2020), hypopharyngeal squamous cell carcinoma (Zhang et al., 2019), prostate cancer (Hu et al., 2019), breast cancer (Ding et al., 2019), and CRC (Liu et al., 2019b). BTF3 initiates transcription by binding to promoter elements, such as TATA box and CAAT box sequences, in the promoter region (Cavallini et al., 1988; Kanno et al., 1992). Due to alternative splicing, BTF3 is present in two different isoforms, BTF3a and BTF3b. BTF3a is the transcriptionally active form of BTF3, while the isoform lacking the first 44 amino acids of the BTF3a N-terminus, BTF3b, is transcriptionally inactive (Parvin et al., 1994). Interestingly, two distinct roles of BTF3 in cancer have been reported. On the one hand, it serves as a transcriptional regulator by targeting the estrogen receptor (ER; Ding et al., 2019) and tumor associated genes (Kusumawidjaja et al., 2007). On the other hand, it is associated with NAC subunit alpha (NACA). The binding of BTF3 with NACA prevents inappropriate targeting of non-secretory nascent polypeptides produced by ribosomes to the endoplasmic reticulum (Koplin et al., 2010). The NAC complex is linked to protein ubiquitination (Panasenko et al., 2009) and proteolysis (Kirstein-Miles et al., 2013) and has been reported to have a related role with BTF3, such as stabilizing BMI1 (Hu et al., 2019) and binding to the N-terminal domain of ER (Green et al., 2007). In our previous study, we reported the oncogenic function of BTF3 in CRC cell lines (Li et al., 2019). According to Liu et al. (2019b), reduced MAD2L2, MCM3, and PLK1 activity may be involved. However, the direct targets of BTF3 as a transcription factor and part of the NAC complex, and the reason for its elevated expression in CRC, remain obscure.

In this study, we first identified the transcriptional targets of BTF3 by applying combined RNA-Seq and chromatin immunoprecipitation (ChIP)-Seq analysis. Thereafter, we conducted immunoprecipitation (IP)-MS and E3 ubiquitin ligase analysis to identify potential interacting targets of BTF3-NACA. Finally, miRNAs targeting BTF3 were predicted and validated. This study provides a comprehensive basis for understanding the oncogenic mechanism of BTF3 in CRC.

## Materials and Methods

### Cell Culture

The colon cancer HT-29 cell line was purchased from the American Type Culture Collection (Manassas, VA, United States). All cells were grown in complete Dulbecco’s Modified Eagle’s Medium (DMEM; HyClone; GE Healthcare Life Sciences, Logan, UT, United States) supplemented with 10% fetal bovine serum (HyClone; GE Healthcare Life Sciences), and 100 U/ml penicillin and streptomycin (Gibco; Thermo Fisher Scientific, Waltham, MA, United States) and incubated at 37°C in a 5% CO_2_ atmosphere.

### Reverse Transcription Quantitative Polymerase Chain Reaction (RT qPCR)

Total RNA was extracted from BTF3-knockdown cell lines (those transfected with BTF3-targeted shRNA), the empty vector control cell line (vector), and non-transfected cells using an RNeasy mini kit (Qiagen China Co., Ltd., Shanghai, China) according to the manufacturer’s protocol. cDNA was generated by reverse transcription of 1-μg aliquots of RNA using the Takara PrimeScript RT Reagent kit (Takara Biotechnology Co., Ltd., Dalian, China) according to the manufacturer’s protocol. cDNA was used for qPCR using the SYBR Premix Ex Taq kit (Takara Biotechnology Co., Ltd.) on a CFX96 instrument qPCR system (Bio Rad Laboratories, Inc., Hercules, CA, United States). PCR was performed according to the manufacturer’s instructions. Initial denaturation was at 95°C for 10 min followed by 30 cycles at 95°C for 1 min, annealing at 53°C for 1 min, extension at 72°C for 1 min, and final extension at 72°C for 5 min. All expression data were normalized to β-actin levels using the 2-ΔΔCq method. Primer sequences were as follows: β-actin, 5′-CGAGCGCGGCTACAGCT-3′ (forward) and 5′-TCCTTAATGTCACGCACGATTT-3′ (reverse); and BTF3 5′-AGCTTGGTGCGGATAGTCTGA-3′ (forward) and 5′-GTGCTTTTCCATCCACAGATTG-3′ (reverse).

### Cell Counting Kit-8 Assay

Cellular viability was determined by the Cell Counting Kit-8 (CCK8; Beyotime Biotechnology). Briefly, 100 μl of H1299 lung cancer cells per well was plated into 96-well plates. After treatment with corresponding reagents, 10 μl of CCK8 solution was incubated with cell medium for 2 h at 37°C. The absorbance of each well was detected at 450 nm by Multiskan FC Microplate spectrophotometer.

### Transwell Assay

Cell migration assay was performed using a modified Boyden chamber plate with 8-μm pore size polycarbonate membrane filters (Corning Incorporated, Corning, NY, United States). ShBTF3- and empty vector-transfected HT-29 cells, along with non-transfected HT-29 cells, were incubated at 37°C in DMEM for 6 h. Subsequently, 1 × 10^4^ cells from each group and cell type were added to the upper part of the Boyden chamber, and the bottom chamber was filled with DMEM containing 20% serum. Cells were allowed to migrate to the underside of the membrane during incubation for 48 h at 37°C. Next, cells on the membrane filter were fixed with 4% paraformaldehyde and stained with 0.05% Giemsa (Sigma-Aldrich; Merck KGaA). The migration index was defined as the number of cells that migrated to the membrane filter by cell counting in at least three random fields per filter using a light microscope (magnification, ×200).

### Wound Healing Assay

Six-well plates were chosen for the wound healing assay. When HT-29 cells were cultured for 24 h after transfection, a plastic pipette tip was used to scratch a line across the cell surface in each plate. The remaining cells were washed three times with PBS to remove the floating cells and debris. Images of the healing process were digitally captured 0 and 24 h after wounding.

### Nude Mouse Xenograft Model

Four-week-old nude female mice were obtained from Slaccas Company. A total of 2 × 10^6^ HT-29 cells of each group were subcutaneously injected 12 h after transfection into mice. For the same cell line, the BTF3 knockdown and normal control groups were implanted into the left posterior flank with the knockdown group in the right of the same mouse. Tumor size was measured every week by calipers. Three weeks later, mice were euthanized, and tumors were statistically analyzed. This study was approved by the Ethics Committees of Changhai Hospital (approval no. 20171001089).

### RNA Sequencing Analysis

We utilized a quartile normalization algorithm to subtract and correct the background. Then, we used the R software Limma package to screen differentially expressed genes (DEGs) by filtering *p* value of Student’s *t*-test and fold change (FC; Diboun et al., 2006). Finally, with a threshold of *p* value <0.05 and absolute value of FC >2, a volcano plot was created using the R software ggplot2 package to identify DEGs with statistical significance between two groups. Hierarchical clustering and combined analyses were performed to identify DEGs. Gene Ontology (GO) enrichment analyses of differentially expressed mRNAs were implemented with EnrichR (Kuleshov et al., 2016). GO terms, including Molecular Function, Biological Processes, and Cellular Components, with *p* values less than 0.05 were considered significantly enriched by DEGs. Kyoto Encyclopedia of Genes and Genomes (KEGG) is a database resource for understanding high-level functions and effects of biological systems.^1^ Cytoscape (version 3.40) was used to visualize the protein–protein interaction (PPI) relationships conducted by String database.^2^

### Gene Expression and Tumor Infiltrate Correlation Analysis

Expression of BTF3 and CHD1L in The Cancer Genome Atlas (TCGA), colon adenocarcinoma (COAD), and rectum adenocarcinoma (READ) datasets were conducted using Gene Expression Profiling Interactive Analysis (GEPIA)^3^ (Tang et al., 2017). Expression of miR-497-5p in Gene Expression Omnibus (GEO) datasets of GSE128446, GSE81581 (Sayagues et al., 2016), and GSE35982 (Fu et al., 2012) were analyzed with the R software Limma package. Gene expression correlation analysis of tumor infiltrates was conducted using TIMER^4^ (Li T. et al., 2017).

### ChIP Sequencing Analysis

HT29 cells were treated with 1% formaldehyde and then quenched with glycine for 5 min at room temperature. ChIP assays were performed using a chromatin IP kit (Cell Signaling Technology, Danvers, MA, United States) according to the manufacturer’s instructions. To perform ChIP analysis of BTF3 binding to the CHD1L promoter, the transcriptional start site was identified using the UCSC Genome browser.^5^ The analysis was conducted and is shown with IGV (Integrative Genomics Viewer).

### Immunoprecipitation

Immunoprecipitation assays were performed using a Pierce Direct Magnetic IP/Co-IP kit (Thermo Scientific, Rockford, IL, United States) according to the manufacturer’s instructions. Briefly, cells were lysed with IP lysis buffer, and protein levels were quantified. Twenty-five microliters of beads coupled with 5 μg of BTF3 antibody were added into 500-μl lysis solutions (1–2 mg/ml) and incubated overnight at 4°C. Eluted proteins were subjected to mass spectrometry analysis using LC–MS/MS (PTM Biolabs, China). IgG (Cell Signaling Technology, Danvers, MA, United States) was used as a negative control.

### Dual Luciferase Reporter Assay

Luciferase reporters were generated based on the psiCHECK2 vector (Promega). The complete 3′UTR of BTF3 mRNA, which included the predicted miR-497-5p binding sites, was PCR amplified and cloned into the psiCHECK2 vector. According to the manufacturer’s guidelines, luciferase reporter genes were co-transfected with miR-497-5p mimics and miR-NC into HT29 cells using Lipofectamine 2000. Dual-Luciferase Reporter Assay System (Promega) and Infinite M200 PRO microplate reader (Tecan) were used to measure the relative luciferase activity.

### Statistical Analysis

All results are expressed as the mean ± standard error of the mean (SEM) and were analyzed using the statistical software SPSS 16.0 (SPSS Inc., Chicago, IL, United States). Chi-square test was performed to assess associations between BTF3 expression and CRC clinicopathological parameters. The Kaplan–Meier plot was performed for survival analysis, and the log-rank test was used to estimate differences in survival. A *p* value <0.05 was considered statistically significant.

## Results

### The Oncogenic Role and Potential Pathways of BTF3 in CRC

**FIGURE 1:**
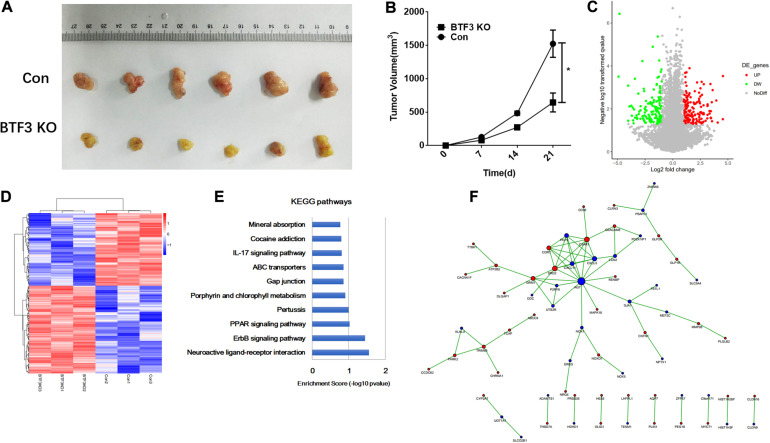
Oncogenic role and potential pathways of BTF3 in colorectal cancer. **(A)** BTF3 knockdown attenuated colorectal cancer proliferation in a xenograft nude mouse model. **(B)** Statistical analysis showing tumor volume in BTF3 knockdown and control groups. **(C)** Volcano plot of DEGs in BTF3 knockdown HT29 cells. **(D)** Heatmap of DEG expression in BTF3 knockdown and control groups. **(E)** Functional enrichment of the top 10 pathways in the BTF3 knockdown group. **(F)** Protein–protein interaction network of DEGs based on the String database. Increased DEGs are shown in red nodes, and decreased DEGs are shown in blue nodes. The larger the node, the higher centrality the gene in the network.

The function of BTF3 in CRC was investigated *in vivo* using a nude mouse xenograft model. As shown in Figure 1A, BTF3 knockdown HT29 cells exhibited reduced tumor size (42.3%, *p* < 0.05, *N* = 6), compared to the control group. Tumor volume was analyzed and is shown in Figure 1B. Consistent with *in vitro* results in our previous research, the *in vivo* phenotype supported the conclusion that BTF3 acts as an oncogene in CRC. Thereafter, BTF3 knockdown and vector-transfected HT29 cells were applied for RNA sequencing and bioinformatics analysis. In total, we identified 292 significantly DEGs with the criteria of expression FC >2 or <0.5 and *p* value <0.05 (Supplementary Table 1). A volcano plot (Figure 1C) and hierarchical clustered heatmap (Figure 1D) were utilized to portray the DEGs. Last, the function of DEGs was analyzed, and the top 10 enriched KEGG pathways are shown in Figure 1E. Neuroactive ligand–receptor interaction, ErbB2, and PPAR were the most significant items. Furthermore, PPI analysis enriched a network composed of AGT, CXCL5, CXCL11, C3AR1, CD36, and so on (Figure 1F).

### BTF3 Activates CHD1L Transcription in CRC

**FIGURE 2:**
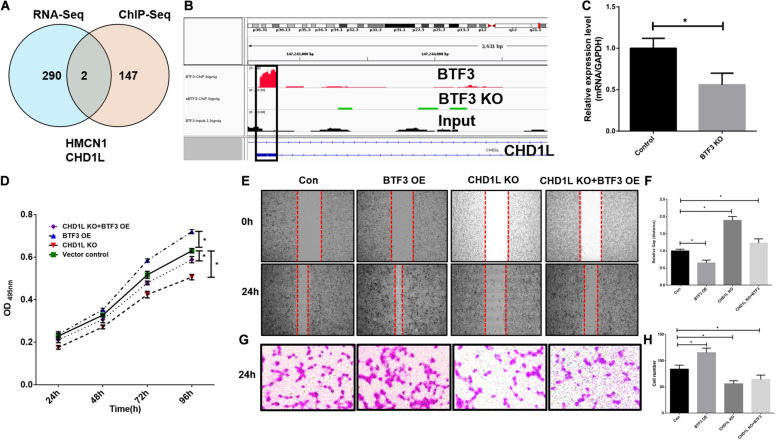
CHD1L is a potential transcriptional target of BTF3 in colorectal cancer. **(A)** Venn diagram showing two common genes, HMCN1 and CHD1L, between significant DEGs with expression and BTF3 binding peak differences. **(B)** ChIP peaks in the CHD1L gene promoter region in BTF3 control, BTF3 knockdown, and input groups. **(C)** Expression of CHD1L mRNA expression in BTF3 knockdown groups. **(D)** CCK8 showing the proliferation effect of BTF3 and CHD1L on HT29 cells. **(E,F)** Would healing assay showing the migration effect of BTF3 and CHD1L on HT29 cells. **(G,H)** Transwell assay showing the invasion effect of BTF3 and CHD1L on HT29 cells. *P < 0.05.

**FIGURE 3:**
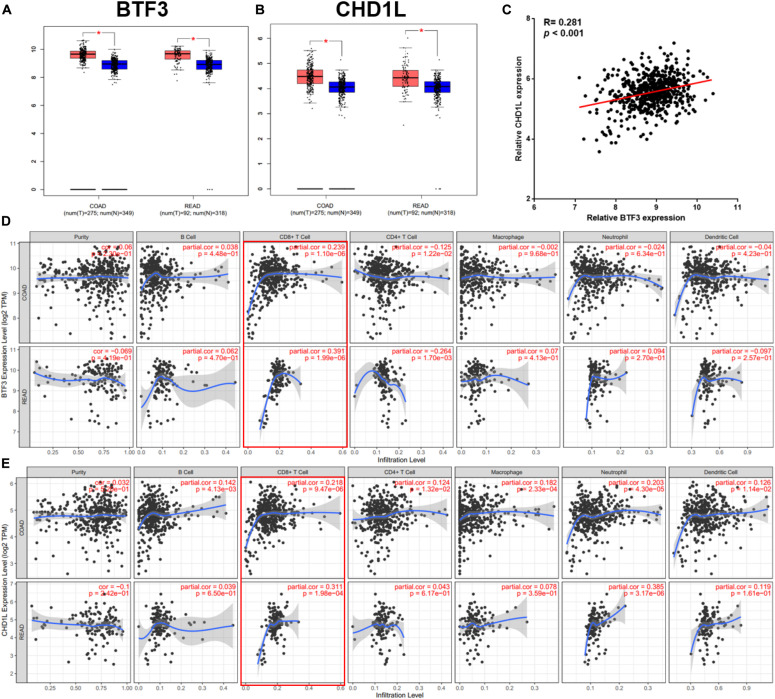
Clinical significance analysis of BTF3 and CHD1L in CRC. **(A)** BTF3 demonstrated significantly higher expression in TCGA COAD and READ datasets. **(B)** CHD1L showed significantly increased expression in TCGA COAD and READ datasets. **(C)** BTF3 was positively correlated with CHD1L expression. **(D)** Correlation analysis of BTF3 with tumor purity and tumor-infiltrating immune cells. **(E)** Correlation analysis of CHD1L with tumor purity and tumor-infiltrating immune cells.

As a transcription factor, BTF3 has been proven to regulate gene expression by binding DNA elements in cancer. Here, to identify potential targets of BTF3, we conducted ChIP sequencing for BTF3 knockdown and control HT29 cells. In total, ChIP-Seq analysis revealed 149 genes with significant differential peaks between the two groups (Supplementary Table 2). As shown in Figure 2A, combined analysis of RNA-Seq and ChIP-Seq results identified two genes with both differential expression and BTF3 binding capability, Hemicentin 1 (HMCN1) and chromodomain helicase DNA binding protein 1 Like (CHD1L). HMCN1 and CHD1L showed increased and decreased expression, respectively, in the BTF3 knockdown group. As a decreased binding peak of BTF3 was located in the promoter region of CHD1L (Figure 2B) and CHD1L has been reported as an oncogene in CRC (Ji et al., 2013) and other cancer types (Chen et al., 2009, 2010, 2011; Li et al., 2013; Liu et al., 2016), we chose CHD1L as a target candidate. qRT-PCR results further revealed that BTF3 knockdown attenuated CHD1L expression (Figure 2C). Next, we examined the function of BTF3 and CHD1L in CRC cell proliferation, invasion, and migration. As shown in Figure 2D, CCK8 assay shows that BTF3 overexpression promoted, while CHD1L knockdown inhibited, HT29 proliferation. In addition, a rescue assay indicated that CHD1L knockdown reduced proliferation capability in BTF3-overexpressing HT29 cells. Similarly, wound scratch assay and Transwell assay results indicated that BTF3 and CHD1L promoted cell migration and invasion (Figures 2E–H), and CHD1L is a target of BTF3. Significantly higher expression of BTF3 and CHD1L was observed in TCGA CRC datasets (Figures 3A,B), and BTF3 showed an evident positive correlation with CHD1L expression. Additionally, correlation analysis of BTF3 and CHD1L expression with clinical pathological parameters was conducted (Figure 3C). As shown in Table 1, BTF3 demonstrated significant correlations with lymphatic invasion and pathologic stage, while CHD1L was correlated with lymph node count and lymphatic invasion. Finally, expression of BTF3 and CHD1L in tumor-infiltrating immune cells was further analyzed, and both genes showed significant correlation with CD8+ T cells (Figures 3D,E), suggesting their potential role in CD8+ T cell regulation. In summary, these results imply that CHD1L is a transcriptional target of BTF3.

**TABLE 1:** Clinical significance analysis of CHD1L, BTF3, and miR-497-5p expression with clinicopathological parameters in CRC.

		CHD1L			BTF3			hsa-miR-497-5p		
Characteristics	No. of Patients(%)	Low	High	*p*	Low	High	*p*	Low	High	*p*
**Gender**				0.077			0.900			0.108
Female	280 (47.2)	151	129		141	139		150	130	
Male	313 (52.8)	146	167		156	157		147	166	
**Age**				0.541			0.428			0.805
<60	169 (28.5)	88	81		89	80		86	83	
≥60	424 (71.5)	209	215		208	216		211	213	
**History of colon polyps**				0.055			0.797			0.976
Yes	159 (31.2)	90	69		81	78		77	82	
No	350 (68.8)	166	184		174	176		170	180	
**Lymph node examined count**				**0.020**			0.929			0.858
Low	299 (52.9)	137	162		154	145		145	154	
High	266 (47.1)	148	118		136	130		131	135	
**Lymphatic invasion**				**0.043**			**0.000**			**0.011**
Yes	217 (40.5)	122	95		130	87		92	125	
No	319 (59.5)	151	168		137	182		171	148	
**Recurrence**				0.078			0.194			0.124
Yes	104 (21.3)	42	62		55	49		46	58	
No	385 (78.7)	193	192		176	209		203	182	
**Non-nodal tumor deposits**				0.430			0.493			0.089
Yes	47 (16.2)	25	22		25	22		18	29	
No	243 (83.8)	114	129		116	127		126	117	
**Pathologic M**				0.963			0.282			**0.033**
0	436 (83.8)	222	214		216	220		232	204	
1	84 (16.2)	43	41		47	37		34	50	
**Pathologic N**				0.732			0.271			**0.013**
0	333 (56.4)	165	168		161	172		182	151	
1–2	257 (43.6)	131	126		136	121		114	143	
**Pathologic T**				0.460			0.251			0.325
1–2	119 (20.1)	56	63		54	65		55	64	
3–4	472 (79.9)	240	232		242	230		242	230	
**Pathologic stage**				0.926			**0.041**			**0.012**
I–II	316 (55.1)	158	158		148	168		174	142	
III–IV	258 (44.9)	130	128		143	115		115	143	
**Preoperative pretreatment CEA level**				0.222			0.425			0.477
Low	194 (50.8)	105	89		108	86		102	92	
High	188 (49.2)	90	98		97	91		92	96	
**Cancer type**				0.659			0.624			0.799
Colon	436 (73.5)	216	220		221	215		217	219	
Rectum	157 (26.5)	81	76		76	81		80	77	

*The bold values are p < 0.05.*

### BTF3 May Regulate p53 Expression Through the E3 Ligase HERC2

**FIGURE 4:**
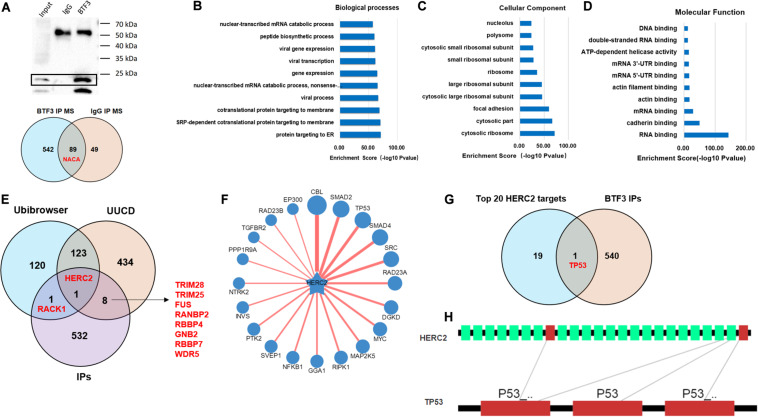
BTF3 may regulate HERC2-mediated p53 ubiquitination in CRC. **(A)** Immunoprecipitation-MS was conducted and revealed 542 BTF3 specific proteins. The biological processes **(B)**, cellular component **(C)**, and molecular function **(D)** of 542 proteins were analyzed, and the top 10 items are shown. **(E)** E3 ligases in Ubibrowser and iUUCD databases were obtained, and HERC2 was enriched in all three lists. **(F)** Substrates of HERC2 were predicted with Ubibrowser. **(G)** Venn diagram of the top 20 predicted HERC2 substrates with 542 BTF3 binding proteins. **(H)** Predicted binding domain of BERC2 with p53.

In addition to the transcription factor role of BTF3, how BTF3 acts as a subunit of NAC was also studied. To identify potential BTF3 binding proteins, BTF3 IP and MS were applied (Figure 4A). Using IgG as a negative control, 542 proteins exhibited BTF3 specific expression (Figure 4A and Supplementary Table 3). Indeed, NACA was identified as binding to BTF3 with different scores. Next, GO annotation and enrichment were conducted. As expected, protein targeting to the ER was the most significant biological process (BP; Figure 4B). For cellular component (CC) analysis, proteins in cytosolic ribosome also supported NACA-related function (Figure 4C). Molecular function (MF) analysis enriched RNA binding as the most evident factor (Figure 4D). Of note, the CC analysis enriched 73 nuclear proteins (e.g., TOP2A, DDX3X, DDX47, CHD7, and ADAR) and MF analysis identified 64 proteins with DNA binding potential (e.g., TOP2A, DDX3X, THRB, MCM7, CHD4, and PHF6), which also supports the transcriptional regulatory role of BTF3. Then, as BTF3 was associated with ubiquitination-mediated proteolysis, we analyzed E3 ligases using two databases, Ubibrowser (Li Y. et al., 2017)^6^ and iUUCD 2.0 (Gao et al., 2013; Zhou et al., 2018). The analyses identified an E3 ubiquitin ligase called HECT and an RLD domain containing E3 ubiquitin protein ligase 2 (HERC2) in all three. Receptor for activated C kinase 1 (RACK1) was another protein in the Ubibrowser and BTF3 interacting protein list. Another eight E3 ligases (TRIM28, TRIM25, FUS, RANBP2, RBBP4, GNB2, RBBP7, and WDR5) were identified by the iUUCD 2.0 database (Figure 4E). Next, the substrates of HERC2 were predicted (Figure 4F), and p53 was the only one predicted in the top 20 protein list (Figure 4G). The interacting domains of HERC2 with p53 are shown in Figure 4H. Taken together, we propose that BTF3 may also play an oncogenic role in CRC via HERC2-mediated p53 ubiquitination and degradation.

### MiR-497 Decreases BTF3 Expression in CRC

**FIGURE 5:**
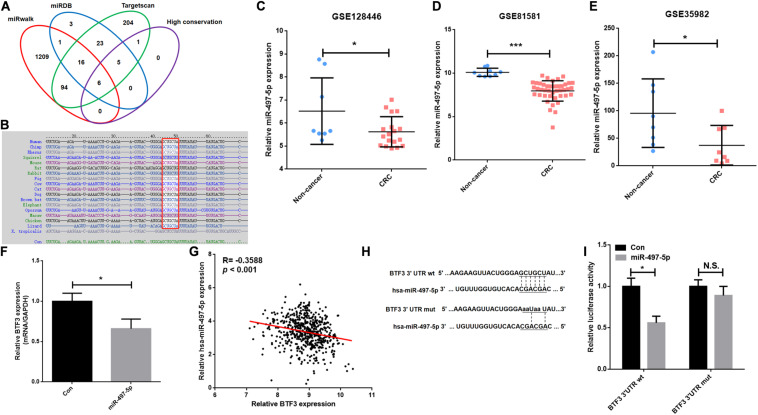
MiR-497-5p is a negative regulator of BTF3 in CRC. **(A)** MiRNAs were predicted with miRwalk, miRDB, and Targetscan with conservation analysis, and six miRNAs were acquired. **(B)** The binding sequence was highly conserved in multiple species. MiR-497-5p was significantly downregulated in CRC samples of GEO datasets, GSE128446 **(C)**, GSE81581 **(D)**, and GSE35982 **(E)**. **(F)** qRT-PCR results of BTF3 expression in miR-497-5p mimics-transfected HT29 cells. **(G)** Significantly negative correlation between miR-497-5p and BTF3 expression in TCGA COAD and READ datasets. **(H)** Predicted binding sequence of miR-497-5p was mutated. **(I)** Luciferase activity in the wild-type group was decreased, while that in the mutation group was not significant. *P < 0.05; N.S., not significant.

A previous study indicated that BTF3 was downregulated by miR-802 in ovarian cancer (Wu et al., 2020). BTF3 expression is significantly increased in CRC, while the role of miRNAs remains obscure. Therefore, we first predicted highly conserved miRNAs using miRwalk, Targetscan, and miRDB (Supplementary Table 4), and as shown in Figures 5A,B, we acquired six miRNA candidates (miR-15a-5p, miR-219a-2-3p, miR-361-5p, miR-497-5p, miR-503-5p, and miR-6838-5p). Then, their expression was analyzed in CRC samples of GEO datasets (GSE128446, GSE81581, and GSE35982). Only one miRNA, miR-497-5p, showed consistent significantly decreased expression in all three datasets (Figures 5C–E). In the next step, we transfected miR-497-5p mimics in HT29 cells, and BTF3 showed significantly decreased expression (Figure 5F). In addition, correlation analysis showed a significantly negative coefficient between miR-497-5p and BTF3 (Figure 5G). Next, the predicted binding sequence of BTF3 3′UTR was mutated (Figure 5H), and a dual luciferase assay was conducted. Indeed, luciferase activity in the wild-type (wt) group was decreased, while that in the mutation group was not significant (Figure 5I). Furthermore, the correlation analysis revealed a significant correlation of miR-497-5p with lymphatic invasion, pathologic M, pathologic N, and pathological stage (Table 1). In summary, these data suggest that elevated expression of BTF3 is caused by decreased expression of miR-497-5p in CRC.

## Discussion

Transcriptional and post-transcriptional gene and pathway dysregulation are important cancer signatures in CRC (Porcellini et al., 2018). BTF3, namely, basic transcription factor 3, has been proven to be an oncogene and hazardous prognosticator in CRC (Liu et al., 2019b). As a transcription factor, BTF3 is believed to regulate gene expression by binding target promoters, and this mechanism has been reported in breast cancer (Porcellini et al., 2018). Here, we refer to this transcription factor characteristic of BTF3 as the transcriptional mechanism, a molecular function primarily occurring in the nucleus. In CRC, the function of BTF3 was first reported to be linked to MAD2L2, MCM3, and PLK1 (Liu et al., 2019b). In addition, the alias name of BTF3 is nascent-polypeptide-associated complex beta (NACB). NAC is highly conserved from yeast to human and is believed to bind nascent peptides from ribosomes. According to S. Rospert et al., multiple hypotheses of NAC molecular functions have been proposed, including transcriptional coactivators and regulators of peptide translocation to ER and mitochondria (Rospert et al., 2002). Recent studies demonstrated that NAC complexes are involved in protein ubiquitination (Panasenko et al., 2009) and proteolysis (Kirstein-Miles et al., 2013), a molecular function primarily occurring in the cytoplasm. As a subunit of NAC, BTF3 has been reported to inhibit ubiquitin-mediated BMI1 degradation (Hu et al., 2019). Based on the BTF3 IP result, cytoplasmic and nuclear proteins were both identified (Figure 4C and Table 1), indicating that both mechanisms may exist for BTF3 in CRC, so we conducted a study on transcriptional and NAC-related mechanism.

To investigate the transcriptional mechanism of BTF3, RNA sequencing and ChIP sequencing were applied, and we identified CHD1L as a target of BTF3. CHD1L is an oncogene in several cancer types, particularly in hepatocellular carcinoma (Chen et al., 2009, 2010, 2011; Li et al., 2013; Liu et al., 2016). CHD1L is also known as amplified in liver cancer protein 1, a chromatin remodeling protein induced in response to DNA damage and a transcriptional regulator. In CRC, CHD1L was reported to promote tumor progression and to predict poor survival (Liu et al., 2019b). Here, we found that CHD1L itself is transcriptionally activated by BTF3. In addition, we observed that, in BTF3 binding proteins, other transcription factors and epigenetic regulators, such as TCF7L2, CHD4, and HDAC2, were identified. Based on the results of this study, we could not rule out the possibility that BTF3 may regulate target gene expression as a transcriptional coactivator by interacting with other transcription factors and epigenetic regulators. In the future, we will explore this possibility in additional studies.

Another finding in this study is the possible mechanism of BTF3 as a regulator of HERC2-mediated p53 ubiquitination and proteolysis. P53 is a well-known tumor suppressor that is regulated by the ubiquitin E3 ligase MDM2 in CRC (Liu et al., 2017; Choi et al., 2018; Zeng et al., 2018). HERC2 is an E3 ligase involved in tumor progression through degradation of cancer-specific substrates (Wu et al., 2010; Izawa et al., 2011; Zhu et al., 2014). Previous studies demonstrated that HERC2 is involved in regulating p53 oligomerization and the MDM2–p53 pathway (Kaustov et al., 2007; Cubillos-Rojas et al., 2014; Garcia-Cano et al., 2020). However, there are still limited studies concerning the role of HERC2 in CRC (Yoo et al., 2011). Here, we found that BTF3 binds HERC2 and p53, and considering the function of BTF3 as an oncogene and BTF3 as a tumor suppressor, we speculated that BTF3 might promote p53 ubiquitination and degradation by recruiting HERC2. To further explore this mechanism, more evidence, including functional analysis of HERC2 and p53 with BTF3, the impact of BTF3 and HERC2 p53 ubiquitination and expression level, co-IP of HERC2 and p53 with BTF3, transcriptome RNA sequencing and ChIP sequencing comparison of p53 and BTF3, etc., would be conducted in a subsequent study.

MicroRNAs (miRNAs) are endogenous single-stranded RNAs of 18–25 nt that are well recognized as post-transcriptional gene expression regulators in CRC (Xuan et al., 2015; Moridikia et al., 2018). In this study, we first predicted six highly conserved miRNAs for BTF3: miR-15a-5p, miR-219a-2-3p, miR-361-5p, miR-497-5p, miR-503-5p, and miR-6838-5p. MiR-15a-5p showed significantly increased expression and was a hazardous prognostic marker in CRC linked to recurrence (Kontos et al., 2017; Al Mustanjid et al., 2020). For miR-219a, miR-219a-5p has been reported to inhibit CRC progression by repressing oncogenic targets (Cheng et al., 2015; Xiong et al., 2015; Huang et al., 2017; Wang et al., 2017), while the function of miR-219a-2-3p in CRC is unclear. Similarly, miR-497-5p also exhibits a tumor-suppressing role in CRC (Wang et al., 2019; Bai et al., 2020; Gharib et al., 2020; Yu and Zhang, 2020). In this study, by expression validation and luciferase assay, we concluded that elevated expression of BTF3 in CRC was mediated by miR-497-5p, whose expression is significantly decreased in CRC.

**FIGURE 6:**
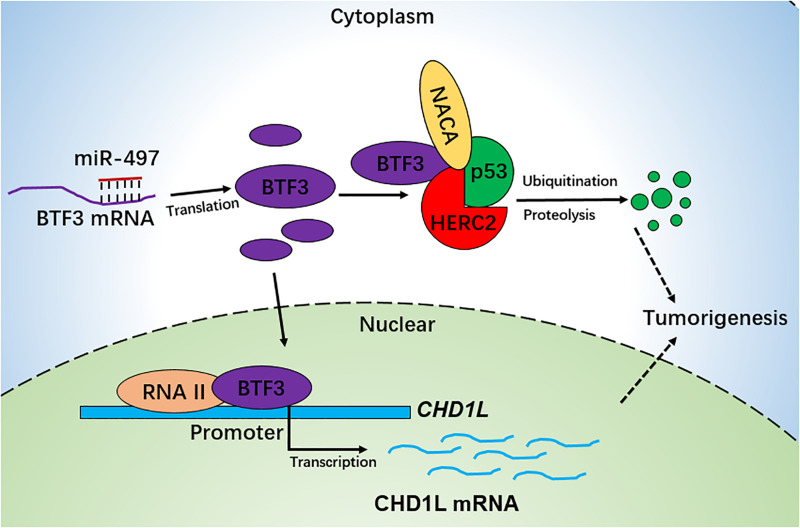
Schematic diagram of BTF3 functional and expression mechanism in CRC. BTF3 is an oncogene in CRC, which may operate using two distinct functional mechanisms. Acting as a transcription factor, BTF3 promotes the transcription and expression of CHD1L, and on the other hand, BTF3 may also inhibit E3 ubiquitin ligase HERC2-mediated p53 degradation. Decreased miR-497-5p expression is responsible for higher levels of BTF3 post-transcriptionally.

Collectively, we conclude that BTF3 is an oncogene in CRC, which may operate using two distinct functional mechanisms. Acting as a transcription factor, BTF3 promotes the transcription and expression of CHD1L. In addition, BTF3 may also inhibit E3 ubiquitin ligase HERC2-mediated p53 degradation. Decreased miR-497-5p expression is responsible for higher levels of BTF3 post-transcriptionally (Figure 6).

## Data Availability Statement

The original contributions presented in the study are included in the article/Supplementary Material, further inquiries can be directed to the corresponding author/s.

## Ethics Statement

The animal study was reviewed and approved by Ethics Committees of Changhai Hospital.

## Author Contributions

HW and JX conceived and designed the experiments. WW performed visualization. GL and HH analyzed the data. MS, YL, and HC prepared figures and/or tables. XL drafted the manuscript. All authors read and approved the final manuscript.

## Conflict of Interest

The authors declare that the research was conducted in the absence of any commercial or financial relationships that could be construed as a potential conflict of interest.

## Footnotes

1 http://www.genome.jp/kegg/

2 https://string-db.org/

3 http://gepia.cancer-pku.cn/

4 https://cistrome.shinyapps.io/timer/

5 http://genome.ucsc.edu

6 http://ubibrowser.nb.org
